# Innate Immunity in Cystic Fibrosis: Varied Effects of CFTR Modulator Therapy on Cell-to-Cell Communication

**DOI:** 10.3390/ijms26062636

**Published:** 2025-03-14

**Authors:** Jennifer Hynes, Clifford C. Taggart, Rabindra Tirouvanziam, Judith A. Coppinger

**Affiliations:** 1 School of Pharmacy and Biomolecular Sciences, RCSI University of Medicine and Health Sciences, D02 YN77 Dublin, Ireland; jenniferhynes24@rcsi.ie; 2 Children’s Health Ireland Translational Research Centre, Children’s Health Ireland at Crumlin, D12 N512 Dublin, Ireland; 3 Airway Innate Immunity Research (AiiR) Group, Wellcome-Wolfson Institute for Experimental Medicine, School of Medicine, Dentistry and Biomedical Sciences, Queen’s University Belfast, Belfast BT9 7BL, UK; c.taggart@qub.ac.uk; 4 Department of Pediatrics, Emory University, Atlanta, GA 30322, USA; tirouvanziam@emory.edu; 5 Center for CF and Airways Disease Research, Children's Healthcare of Atlanta, Atlanta, GA 30322, USA

**Keywords:** bacteria, CFTR, epithelium, EV, HEMT, macrophage, neutrophil

## Abstract

Cystic Fibrosis (CF) is a life-shortening, multi-organ disease caused by mutations in the CF transmembrane conductance regulator (CFTR) gene. Prominent clinical features of CF take place in the lung, hallmarked by cycles of bacterial infection and a dysfunctional inflammatory airway response, leading to eventual respiratory failure. Bidirectional crosstalk between epithelial cells, leukocytes (e.g., neutrophils, macrophages) and bacteria via release of intra-cellular mediators is key to driving inflammation in CF airways. In recent years, a highly effective combination of therapeutics targeting the CFTR defect have revolutionized treatment in CF. Despite these advancements and due to the complexity of the immune response in the CF airway, the full impact of highly effective modulator therapy (HEMT) on airway inflammation is not fully determined. This review provides the evidence to date on crosstalk mechanisms between host epithelium, leukocytes and bacteria and examines the effect of HEMT on both soluble and membrane-derived immune mediators in clinical samples. The varied effects of HEMT on expression of key proteases, cytokines and extracellular vesicles (EVs) in relation to clinical parameters is assessed. Advances in treatment with HEMT have shown potential in dampening the chronic inflammatory response in CF airways. However, to fully quell inflammation and maximize lung tissue resilience, further interventions may be necessary. Exploring the effects of HEMT on key immune mediators paves the way for identifying new anti-inflammatory approaches targeting host immune cell interactions, such as EV-directed lung therapies.

## 1. Introduction

Cystic Fibrosis (CF) is a genetic disease caused by mutations in the CF transmembrane conductance regulator (CFTR) gene, resulting in a dysfunctional or absent CFTR protein on the apical membrane of epithelial cells in all exocrine organs in the body (e.g., sweat glands, salivary glands, exocrine pancreas, genital tract, gut and lung). Through direct and indirect mechanisms, CFTR defects lead to complex organ pathophysiology. In the lungs, CF is associated with a triad of muco-obstruction, inflammation and infection, highlighting significant defects in host defence.

Defects in CF lung host defenses are multifaceted, encompassing innate vs. adaptive signalling pathways, effector vs. regulatory mechanisms and tissue damaging vs. pro-resolution processes. This complex regulation stems from cooperative mechanisms and crosstalk between [1] host epithelium, [2] host leukocytes (neutrophils and macrophages chiefly), and [3] bacteria. Host–bacterial crosstalk is mediated by exchange of soluble factors and extracellular vesicles (EVs) originating from all cells within the CF airway microenvironment. This short perspective outlines the roles of soluble factors and EVs in modulating immunity in CF, and assesses the potential effects of highly effective CFTR modulator therapy (HEMT, the new standard molecular therapy for CF) on cell–cell communication.

### 1.1. Cystic Fibrosis and CFTR

The CFTR protein transports chloride and bicarbonate ions across epithelial surfaces and functions as a negative regulator of the epithelial sodium (Na+) channel (ENaC). CFTR dysfunction impacts innate immune responses by impairing mucociliary clearance (MCC), and is linked to persistent airway infection and unresolved inflammation, leading to progressive tissue damage and lung function decline [1]. This muco-obstructive/inflammation cascade is mediated by an intrinsic dysfunction of host immune responses in people with CF (PwCF), resulting in a hyper-inflammatory state [2,3,4]. Innate defenses are further altered during exacerbations, resulting in disease flares [5].

CFTR controls, in part, the hydration of the airway surface liquid (ASL) via chloride secretion and inhibition of ENaC-mediated sodium absorption to maintain a healthy ASL. This enables efficient ciliary beating and clearance of luminal material, including incoming pathogens. However, in CF, impaired chloride secretion and excess sodium absorption are thought to alter ASL properties, leaving mucosal surfaces more susceptible to bacterial colonization and unresolved inflammation. In addition, potential acidification of the ASL through reduced CFTR-mediated bicarbonate transport is thought to impair phagocyte function and ciliary beat frequency [6], adding to the burden of uncleared pathogens and muco-inflammatory products within the airways. Furthermore, mucus viscosity is altered by the abundance of free neutrophil-derived actin and DNA [7], as well as increased interleukin (IL)-1α and IL-1β-induced mucin hypersecretion [8,9], making it more difficult to clear from the airways. The cumulative result of these factors (less water, more actin, DNA and mucins) leads to the formation of mucus flakes and plugs and results in the establishment of an intense muco-inflammatory airway environment in CF. Mucus plugging creates local hypoxic regions in CF airways and in combination with increased epithelial cell oxygen consumption driven by ENaC hyperactivation, promotes epithelial cell necrosis [10,11].

It is important to note that the impact of CFTR on MCC is only relevant to the nose and large airways, where cilia actually mediate mucus movement. In the small airways, where CF lung disease starts (during infancy and early childhood) there is no mucociliary system and the formation of discrete flakes on top of the epithelium due to pro-inflammatory signalling, rather than a chloride/hydration effect is believed to trigger local alterations in bacterial clearance, opening the door to colonization [12]. Thus, therapies aimed at immune signalling may be most efficacious in stemming early CF small airway disease, while therapies aimed at improving the mucociliary defect may be more relevant to later stages of the disease, where large airways are involved [13].

### 1.2. Chronic Infection and Inflammation in CF Airways

Both small airway-borne pro-inflammatory environments and large airway-borne muco-obstructive environments contribute to cycles of persistent infection in CF lungs, reflecting altered host lung defenses. However, the altered defenses do not lead to a rapid demise of the lung, as seen in patients with severe bacterial pneumonia following severe flu, SARS-CoV-2 infection, or sepsis. Instead, PwCF develop long-lived airway microbiomes, dominated by opportunistic organisms (notably *Staphylococcus aureus* (*S. aureus)*, and *Pseudomonas aeruginosa* (*P. aeruginosa)*), reflecting host/pathogen crosstalk and mutual adaptations [14] rather than a constant tug-of-war, as sometimes thought. Anomalies in several signalling pathways can contribute to the sustained pro-inflammatory microenvironment in the CF lung. These include abnormal glucose metabolism [15], the phosphatase and tensin homolog 10 complex with CFTR [16], altered miRNA regulation [17], ER stress [18], autophagy [19], and a platelet accessory role [20] leading to myeloid cell recruitment/dysfunction in CF airways. CFTR has also been proposed to directly impact phagosome chlorination, lowering bactericidal capacity in both CF macrophages and neutrophils [21,22].

Neutrophils are key myeloid drivers of the hyper-inflammatory state in CF lungs, contributing to mucosal stress through their release of intracellular components such as reactive oxygen species and proteases [23]. The resulting protease burden exceeds the capacity of antiproteases, leading to tissue damage and bronchiectasis [24,25]. Investigative studies on neutrophil dysfunction in CF suggest neutrophils in CF patients may present defective exocytosis [26] or die prematurely upon recruitment to CF airways, via either secondary necrosis following apoptosis and lack of macrophage efferocytosis, or NETosis [7,27]. However, other studies have shown that airway neutrophils in children and adults with CF largely remain alive [28,29,30] and adopt a fate featuring primary granule release, immunomodulatory activity, and metabolic licensing (termed “GRIM”). GRIM neutrophils actively exocytose their content, leading to high extracellular levels of neutrophil elastase (NE), a hallmark of CF. Neutrophil-derived NE disarms macrophages via multiple mechanisms [31], including inhibition of macrophages, via induction of PD-1 checkpoint signalling [32]. Additionally, GRIM neutrophils have been reported to suppress T-cell function [33] and undergo active repression of bacterial killing [34] explaining the infection-tolerant state in CF airways.

While neutrophilic dysfunction strongly contributes to the progression of airway disease in PwCF, other immune cells also play crucial roles in modulating CF hyperinflammation. Monocytes (Crc2) drive tissue damage through pathogenic transforming growth factor β (TGF-β) signalling in the CF lung, facilitating neutrophil recruitment [35]. CF macrophages have been shown to produce elevated levels of pro-inflammatory cytokines (Section 1.3.1) due to intrinsic CFTR dysfunction, dysregulated signalling and reduced phagocytic activity leading to defective bacterial killing [22,36]. Macrophages in CF have also been shown to have altered expression of molecules involved in antigen presentation and T cell activation [37]. T cells contribute to chronic inflammation in CF with a skewed Th17 and Th2-mediated cytokine response reported, increasing infection susceptibility [38]. Furthermore, in PwCF with chronic *P. aeruginosa* infection, decreased levels of regulatory T cells (Tregs) have been shown to correlate with reduced lung function [39].

### 1.3. Key Soluble Factors and EVs Modulating Innste Immunity in CF

#### 1.3.1. Immune Mediators and Growth Factors

CFTR-mediated epithelial and leukocyte dysfunction results in aberrant mediator production, leading to increased microbial infection despite enhanced neutrophil recruitment [40,41]. Mediators found in excess in the CF airway lumen include both host molecules like tumour necrosis factor (TNF)-α and pathogen-associated molecular patterns (PAMPs) such as bacterial lipopolysaccharide (LPS), which ligate cognate receptors on host cells (e.g., Toll-like receptor (TLR)-4). Among host mediators, several studies have emphasized the impact of IL-8 (a.k.a., CXCL8) in CF airways, which, when released from epithelial cells and macrophages, serves as a potent chemoattractant to sustain recruitment of short-lived neutrophils to CF airways [42,43]. Inflammasome activation in host cells is also thought to contribute to the chronic recruitment of neutrophils to CF airways, via both IL-1α from the stressed epithelium and IL-1β from activated neutrophils themselves [11,44]. Furthermore, airway macrophages normally acting as sentinels of the lung can secrete pro-inflammatory cytokines to recruit neutrophils [45]. Airway macrophages have been associated with elevated levels of pro-inflammatory cytokines, such as TNF-α and IL-1β, in the CF airway [37]. Enhanced recruitment of neutrophils may also stem from a reduced expression of pro-resolution mediators, such as IL-10 [46] and resolvins [47].

#### 1.3.2. Proteases

The protease burden in the CF airways is elevated beyond the control of endogenous antiprotease inhibitors, resulting in the degradation of extracellular matrix (ECM) components, which disrupts bacterial clearance and induces inflammation [48,49]. The elevated levels of NE found in bronchoalveolar lavage fluid (BALF, a reflection of small airway physiology) has been shown to be a risk factor for early bronchiectasis in CF infants [4]. NE can cleave and inactivate, or activate, a range of important inflammatory mediators in the CF lung including the neutrophil CXCR1 receptor, the phagocytic receptors CD14 and CD16 to disable neutrophil bacterial killing, and two of its own inhibitors, secretory leukocyte protease inhibitor and elafin [50,51,52,53]. CXCR1-derived fragments from NE degradation bind to TLR-2, stimulating IL-8 release and subsequent neutrophil recruitment, perpetuating the vicious inflammatory cycle [54,55,56].

Furthermore, matrix metalloproteinases (MMPs), which are capable of degrading the protein components of the ECM, are elevated in the CF airway and are implicated in airway remodelling [57]. MMP-8, MMP-9, and the tissue inhibitor of metalloproteinase-1 (TIMP-1) are all upregulated in CF BALF and sputum, compared to their healthy control counterparts [57,58,59]. Sputum MMP-9 levels negatively correlate with lung function in both paediatric and adult PwCF. A study of BALF from children with CF enrolled in the AREST CF study showed that MMP-9 activation increased with free NE and that MMP-9/TIMP-1 ratios strongly correlated with bronchiectasis progression in the preceding year [49,60,61,62]. More recently, the protease cathepsin S (CatS) has been shown to be elevated in both paediatric and adult PwCF [63,64]. CatS can degrade important host defense proteins such as lactoferrin, LL-37 and β-defensins and treatment with a CatS inhibitor in a transgenic mouse model with CF-like lung disease (βENaC-overexpressing mice) reduced lung inflammation, mucus obstruction and lung damage [64,65].

#### 1.3.3. Extracellular Vesicles (EVs)

EVs are small vesicles (generally ranging from 50–200 nm in diameter) released from all living cells, enriched with protein and RNA cargo, that mediate communication between cells. The ability of EVs to bear disease-specific signatures is increasingly recognized across several diseases [66]. We recently discovered that EVs are produced in unusually large amounts by CF airway cells, and can drive neutrophil migration into the airways [67,68]. Of further interest, CF airway neutrophil-derived EVs modulate the phenotype of naive neutrophils [69] and promote feed-forward inflammasome signalling in CF airway epithelial cells [68]. Cross-kingdom signalling via EVs is also recognized in the context of CF airways with bacterial EVs having a significant impact on host epithelial responses [70,71]. However, the reciprocal responses of bacterial cells to host-derived EVs remains to be investigated.

## 2. Highly Effective CFTR Modulators (HEMT)

### 2.1. HEMT Classification

The development of HEMTs as a new class of CF therapy targeting the underlying CFTR defect represents a significant progression in the management of the disease [72]. Currently, five types of HEMTs have been developed: potentiators, correctors, stabilizers, read-through agents, and amplifiers. Potentiators were the first class of CFTR modulators to be developed (e.g., ivacaftor [Kalydeco, VX-770]) acting on CFTR channels at the cell surface to increase gating (open probability) and conductance of ions. Ivacaftor was reported to have significant benefits in those affected by the G551D CFTR mutation in two large multicentric trials [73,74]. Correctors were then identified (e.g., lumacaftor [VX-809]), initially showing favourable results in PwCF bearing the most common CFTR mutation (F508del) in a phase II trial by increasing transport of CFTR to the cell surface [75]. Orkambi, a combination of ivacaftor and lumacaftor, was developed to correct both protein trafficking and channel gating abnormalities. Wainwright et al. assessed the efficacy of this combination therapy in PwCF aged 12 and older, homozygous for the F508del mutation, and showed significant improvements in lung function, as indicated by FEV1 [76]. Another CFTR corrector, tezacaftor, was shown to be well-tolerated and efficacious in patients with the F508del mutation, with even greater improvements observed in FEV_1_ when delivered in combination with ivacaftor [77].

Following the FDA approval of the triple combination therapy Trikafta/Kaftrio (elexacaftor-tezacaftor-ivacaftor, ETI) in 2019, approximately 90% of PwCF have genotypes amenable to HEMT treatment including those with F508del mutations [78,79]. A new combination therapy, Alyftrek (vanzacaftor-tezacaftor-deutivacaftor, VTD), was then developed as a once-a-day alternative to ETI with promising results [80] and has since gained approval from the US FDA. ETI therapy has led to significant improvements in CF lung disease across various measures, with multiple studies demonstrating its efficacy in improving lung function, indicated by parameters such as lung clearance index (LCI), FEV_1_, pulmonary exacerbation rate and sweat chloride concentration (SCC). The drug has also been shown to enhance overall quality of life, with similar effects expected with VTD.

**Figure 1:**
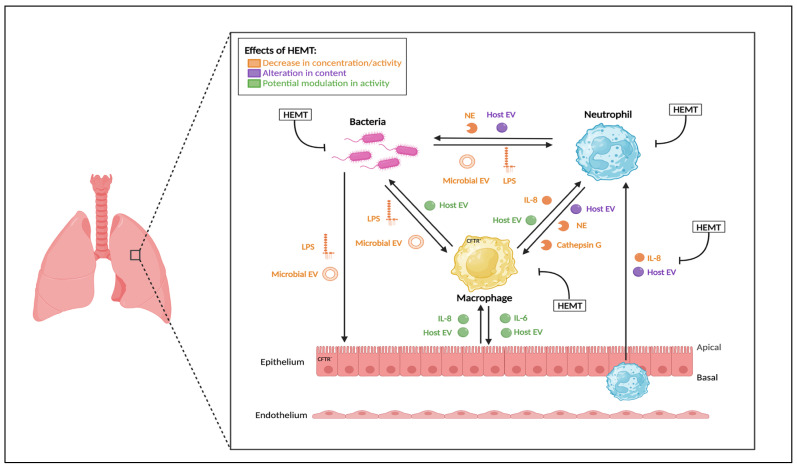
**The potential effect of HEMT on the CF lung microenvironment**. The CF airway hosts complex interkingdom bidirectional interactions between epithelial cells, neutrophils, macrophages and bacteria. Bacteria release lipopolysaccharide (LPS) and outer membrane vesicles (OMVs), which can activate a CF host response via epithelial cells, macrophages and neutrophils. Increased levels of cytokines like IL-8 and vesicles (EVs) drive increased neutrophil recruitment to the CF airway. CF airway neutrophils exhibiting GRIM phenotypes release further vesicles and proteases including neutrophil elastase (NE). NE can modulate resident macrophage responses to bacterial challenge, further driving both inflammation and tissue damage in the CF lung. HEMT acts as a potential modulator of these interactions influencing immune cell activity, epithelial responses and bacterial burden. Created in BioRender. Hynes, J. (2025). https://BioRender.com/d84b289.

Despite many reported clinical benefits of HEMT, there has been varying reports of the effects of HEMT to date on CF airway immunity [81] with several studies reporting dampened but persistent inflammation in the CF lung. The potential effects of HEMT on the CF airway microenvironment is highlighted in Figure 1.

### 2.2. Effects of Monotherapy

Studies investigating the effects of ivacaftor monotherapy on inflammation have produced varying results. One study examining adult PwCF on ivacaftor treatment observed no significant changes in inflammatory cytokines (IL-8 and IL-1β) or proteases (such as NE and SLP1) in the sputum, despite measurable improvements in FEV_1_ and SCC [82]. Similar findings were reported in a cohort of paediatric PwCF, with no significant changes in BALF inflammatory biomarkers (IL-8, NE) following treatment with ivacaftor [83]. Another study focused on ivacaftor found limited and transient reduction in the levels of *P. aeruginosa* and *S. aureus* in the sputum of PwCF [84].

However, other studies investigating ivacaftor reported significant clinical and inflammatory improvements post-treatment. Hisert et al. observed reductions in expression of inflammatory cytokines (NE, IL-8) in sputum along with improved FEV1, mucus clearance and reduced bacterial concentrations [85]. A long-term study associated with the UK registry demonstrated reduced lung infection by key CF pathogens, including *P. aeruginosa*, with ivacaftor treatment [86]. Furthermore, a study examining the effect of ivacaftor on systemic inflammation observed a reduction in blood inflammatory markers, including HMGB1 and calprotectin, alongside clinical enhancements in FEV1 and weight with treatment [87]. Additionally, a non-significant reduction in EVs was observed in a small number of PwCF one year after commencing treatment [67].

### 2.3. Effect of Dual Therapy

Studies investigating dual modulator therapy saw some improvements in both markers of inflammation and clinical parameters. Wainwright et al. demonstrated reduced numbers of bacterial-induced exacerbations in a large study of PwCF along with improved FEV1 with ivacaftor/lumacaftor (IVA/LUM) treatment [76]. Other studies have focused more on inflammation with dual therapy. For example, an in vitro study demonstrated that CFTR functional rescue with IVA/LUM greatly reduced CXCL8 (as well as CXCL1 and CXCL2) transcripts and p38 MAPK phosphorylation in epithelial cells exposed to *P. aeruginosa* [88]. A study in adult PwCF showed reductions in inflammatory markers with dual combinations of CFTR modulators. Specifically, peripheral blood mononucleated cell releases of IL-18, TNFα and caspase-1 (a central inflammasome enzyme) were decreased in PwCF on IVA/LUM and ivacaftor/tezacaftor (IVA/TEZ), while decreased IL-1β release and pro-IL-1β mRNA levels were observed with IVA/TEZ alone [89]. Another study corroborated these findings by showing reduced IL-1β levels in sputum of F508del homozygous PwCF on IVA/LUM, and a reduction in total bacterial load [90]. Furthermore, Arooj et al. detected a significant reduction in plasma cytokines including IL-8, TNF-α and IL-1β levels, but not IL-6, after 12 months of IVA/LUM treatment [91]. However, not all investigations demonstrated improvements in lung function and associated inflammation. A study in children with CF aged 6–11 examining the use of IVA/LUM over two years found worsening bronchiectasis scores [92]. Another study examining the effects of IVA/LUM on airway microbiota in PwCF (>12y) with existing *P. aeruginosa* infections did not observe significant changes in bacterial diversities, pathogen abundances, or the inflammatory marker calprotectin [93].

### 2.4. Effect of Triple Therapy

Triple therapy (so far limited to ETI in scientific reports) has exhibited substantial clinical benefits but varying effects on inflammatory signalling. Lepissier et al. reported significant reductions in inflammatory cytokines such as IL-8 in sputum, accompanied by clinical improvements, with ETI treatment [94]. Similar findings were reported by Maher et al. who observed a reduction in the expression of inflammatory molecules, such as S100-A8, following ETI treatment [95]. However, the effects of CFTR modulator therapy on neutrophil serine protease (NSP) levels, such as NE, have yielded contrasting findings. Maher et al. did not observe significantly reduced NSP levels (NE, CatG, PR3) or increased levels of alpha 1 antitrypsin in the sputum of PwCF [95]. Trappe et al. in fact, demonstrated a decrease in expression of the protease inhibitor alpha-1 antitrypsin in EVs isolated from sputum from PwCF one year after commencing Kaftrio [96]. Schaupp et al. demonstrated that ETI treatment over 12 months resulted in decreases in proteases, free NE activity, CatG and PR3 and *P. aeruginosa* levels in CF sputum but without eliminating NSP activity in this group [97]. Casey et al. reported similar anti-inflammatory effects of ETI, including decreased activity of neutrophil enzymes (e.g., NE and proteinase 3), which coincided with sustained improvements in lung function and reduced sputum production [98]. In this study, the authors observed that airway inflammation in PwCF treated with ETI was not eliminated but rather returned to the non-CF bronchiectasis range, supporting the concept that for PwCF with established lung disease, restoration of CFTR results in disease modification and a phenotype shift rather than a definitive cure [98]. Furthermore, several studies have demonstrated a reduction, but not elimination in the prevalence of *S. aureus* and *P. aeruginosa* in PwCF after initiation of CFTR modulator therapy, including a recent study with data from 1092 PwCF [99]. Hence, adjunct therapies addressing chronic adaptations such as bacterial tolerance and sustained neutrophilic inflammation are likely needed to reach full recovery in PwCF, beyond the correction of CFTR dysfunction. A summary of our findings and some additional studies are presented in Table 1.

**Table 1:** Effects of HEMT modalities on inflammatory signalling in CF. The varied effects of CFTR modulator therapy (mono, dual and triple) on immune mediators in relation to clinical outcome are highlighted in this table.

Modulator	Sample	Effect of Modulator on Inflammation	Outcome	Reference
**Monotherapy** **(Ivacaftor only)**	Sputum	No significant changes in levels of inflammatory cytokines (e.g., NE, IL-8, IL-1β) in sputum	Clinical improvements in FEV_1_ and SCC.	[82]
	BAL	No significant changes in NE positivity, IL-8, or absolute neutrophil count in BAL	Retrospective but clinical improvements in lung function previously reported for these cohorts	[83]
	Sputum	↓ levels of inflammatory cytokines (e.g., NE, IL-8, IL-1β) in sputum	Clinical improvements in FEV_1_ and mucus plugging, as well as reduced bacterial concentrations in sputum	[85]
	Blood	↓ levels of a pro-inflammatory mediator (HMGB-1) and neutrophilic inflammatory markers (calprotectin and G-CSF) in circulation	Clinical improvements in FEV_1_, weight, BMI, and SCC	[87]
	Sputum	No changes in sputum inflammatory markers including NE	Clinical improvement in FEV_1_ and MCC	[100]
	Nasal lavage	↓ levels of IL-1β, IL-6 and IL-8 in nasal lavage	Significant clinical improvements in FEV_1_ and SCC	[101]
**Dual Therapy**	Lung cells	IVA/LUM significantly ↓ CXCL8, CXCL1 and CXCL2 transcripts in response to *P. aeruginosa* exposure in primary HBE cells	Potential ↓ lung inflammation but no clinical parameters measured	[88]
	Blood	↓ in IL-18 with IVA/LUM and IVA/TEZ in CF monocytes/serum/PBMCs, but ↓ in IL-1β levels only found with IVA/TEZ	No significant changes in clinical parameters, consistent with stability in disease, rather than decline in health	[89]
	Sputum	↓ levels of IL-1β in sputum with IVA/LUM, but no change in other inflammatory mediators, e.g., IL-6, IL-8, TNFα, and NE activity	No change in FEV_1_, but improvements in LCI, MRI morphology, perfusion score and total bacterial load	[90]
	Blood	↓ in IL-1β, IL-8 and TNFα levels in plasma with IVA/LUM	Clinical improvement in FEV_1_ and SCC	[91]
	Blood	↓ in WBC counts and serum CRP levels with IVA/LUM	Improvements in FEV_1_, SCC and BMI	[102]
**Triple Therapy (ETI)**	Sputum Blood	↓ in levels of inflammatory cytokines (e.g., NE, IL-8, IL-1β) in sputum in parallel to CRP and PMN count in blood, indicating blunted neutrophil-derived inflammation	Clinical improvements in FEV_1_ and body weight, with some patients also presenting with decreased SCC	[94]
	Sputum	↓ reduction in neutrophil-derived proteins (eg. S100-A8) but not proteases (eg. NE) in sputum, but overall improved balance of harmful/beneficial proteins	Clinical improvements in FEV_1_	[95]
	Sputum	↓ in expression of protease inhibitor alpha 1 antitrypsin in sputum EVs	Clinical improvements in lung function and FEV_1_	[96]
	Sputum	↓ IL-8 at 3 months and free NE at all timepoints (1, 3, 1 2 months) in sputum	Improvements in FEV_1_ and relative abundance of *P. aeruginosa,*	[97]
	Sputum Blood	↓ activity of NE, proteinase 3 and cathepsin G, and ↓ concentrations of IL−1β and IL-8 in sputum. Also restoration of secretory leukoprotease inhibitor levels.	Improvements in FEV_1_, irrespective of the degree of pre-ETI airflow obstruction, sustained at 1 year.	[98]
	Plasma	↓ levels of MIP-3α, GROα and IL-8 (not significant) in plasma	Improvements in FEV_1_ and SCC	[103]
	Blood	No change in cytokine (e.g., IL-1β, IL-8) secretion in monocyte-derived macrophages from either CF or non-CF individuals.	No clinical parameters measured	[104]

## 3. Conclusions and Future Directions

The advent of HEMT has changed the landscape of CF care for the better, with significant improvements in organ function and quality of life for a large majority of PwCF with eligible mutations and access to these CFTR-targeted drugs. While HEMT broadly improves lung function and physiological parameters such as MCC, its effects on underlying pro-inflammatory signalling, while often detectible, are less pronounced. So far, improvements in the expression of immune mediators (e.g., IL-8) and proteases (e.g., NE) have been observed in several studies (Table 1). Select studies have examined the response of immune cell subsets (e.g., CF macrophages) to HEMT [105]. However, more focused studies are needed to assess changes in EV signalling by the host epithelium, neutrophils, macrophages and bacteria under HEMT, as many studies to date have focused on measuring inflammatory mediators in lung fluids and plasma, rather than from individual cell populations. An open question relating to this would be what the relative impact of HEMT is on the host epithelium vs. immune cells in which CFTR expression is known (macrophages) or under question (neutrophils).

Of further note, there has been a larger focus on examining soluble mediators released in to airway fluids but less is known about EVs, which are known to mediate important cell–cell crosstalk in chronic CF airway disease [66,67,68]. EV-directed therapies have been successful in models of lung injury and acute respiratory distress via reprogramming of myeloid cells [106,107]. Additionally, EVs have been shown to dampen the inflammatory signature in subsets of COVID-19 patients, a subject of ongoing clinical trials [108]. EV-directed lung therapy in CF and chronic lung disease with host-directed interventions is also viable, and a developing therapeutic area distinct from antibiotics and typical anti-inflammatory drugs, which can have limited efficacy [109]. In addition to reducing inflammation and bacterial challenge, manipulation of host EVs can reduce tissue repair within the lung, which is of particular significance in the CF population with existing bronchiectasis prior to commencing HEMT.

The advent of a new generation of HEMT will undoubtedly spark a new collection of biomarker studies, providing further insights. Targeted studies on how specific inflammatory cell populations communicate in CF airways in the presence of HEMT should help address current knowledge gaps and pave the way for the development of novel therapies in CF and CFTR-related lung diseases.

## Abbreviations

CF	Cystic Fibrosis
CFTR	Cystic Fibrosis Transmembrane Conductance Regulator
EV	Extracellular Vesicle
HEMT	Highly Effective Modulator Therapy
MCC	Mucociliary Clearance
FEV_1_	Forced Expiratory Volume in 1 s
PwCF	Persons with Cystic Fibrosis
ASL	Airway Surface Liquid
IL-8	Interleukin-8
GRIM	Primary Granule Release, Immunomodulatory Activity, and Metabolic Licensing
NE	Neutrophil Elastase
TNF-α	Tumour Necrosis Factor alpha
PAMP	Pathogen-Associated Molecular Pattern
LPS	Lipopolysaccharide
ECM	Extracellular Matrix
BALF	Bronchoalveolar Lavage Fluid
MMP	Matrix Metalloproteinases
ETI	Elexacaftor-Tezacaftor-Ivacaftor (Trikafta/Kaftrio)
SCC	Sweat Chloride Concentration
